# Functional connectivity of white matter as a biomarker of cognitive decline in Alzheimer’s disease

**DOI:** 10.1371/journal.pone.0240513

**Published:** 2020-10-16

**Authors:** Yurui Gao, Anirban Sengupta, Muwei Li, Zhongliang Zu, Baxter P. Rogers, Adam W. Anderson, Zhaohua Ding, John C. Gore, for the Alzheimer’s Disease Neuroimaging Initiative

**Affiliations:** 1 Institute of Imaging Science, Vanderbilt University Medical Center, Nashville, Tennessee, United States of America; 2 Department of Biomedical Engineering, Vanderbilt University, Nashville, Tennessee, United States of America; 3 Department of Radiology and Radiological Sciences, Vanderbilt University Medical Center, Nashville, Tennessee, United States of America; 4 Department of Electrical Engineering and Computer Science, Vanderbilt University, Nashville, Tennessee, United States of America; University of Rome Tor Vergata, ITALY

## Abstract

**Objective:**

*In vivo* functional changes in white matter during the progression of Alzheimer’s disease (AD) have not been previously reported. Our objectives are to measure changes in white matter functional connectivity (FC) in an elderly population undergoing cognitive decline as AD develops, to establish their relationship to neuropsychological scores of cognitive abilities, and to assess the performance in prediction of AD using white matter FC measures as features.

**Methods:**

Analyses were conducted using resting state functional MRI and neuropsychological data from 383 ADNI participants, including 136 cognitive normal (CN) controls, 46 with significant memory concern, 83 with early mild cognitive impairment (MCI), 37 with MCI, 46 with late MCI, and 35 with AD dementia. FC metrics between segregated white matter tracts and discrete gray matter volumes or between white matter tracts were quantitatively analyzed and characterized, along with their relationships to 6 cognitive measures. Finally, supervised machine learning was implemented on white matter FCs to classify the participants and performance of the classification was evaluated.

**Results:**

Significant decreases in FC measures were found in white matter with prominent, specific, regional deficits appearing in late MCI and AD dementia patients from CN. These changes significantly correlated with neuropsychological measurements of impairments in cognition and memory. The sensitivity and specificity of distinguishing AD dementia and CN using white matter FCs were 0.83 and 0.81 respectively.

**Conclusions and relevance:**

The white matter FC decreased in late MCI and AD dementia patients compared to CN participants, and this decrease was correlated with cognitive measures. White matter FC is valuable in the prediction of AD. All these findings suggest that white matter FC may be a promising avenue for understanding functional impairments in white matter tracts during AD progression.

## Introduction

Alzheimer’s disease (AD) is the most common progressive neurodegenerative disorder, which begins at a pre-symptomatic stage before subjects exhibit increasingly severe cognitive impairments and ultimately, dementia [1, 2]. Histopathological evidence of degeneration during this progression has been observed in human brains in both gray matter (GM) and white matter (WM) [3, 4]. While there has been considerable emphasis on GM changes, pathological alterations of WM post mortem have also been reported not only in late stages of AD (associated with loss of axons and oligodendrocytes [5] and concomitant with vascular abnormalities [6, 7]), but also in earlier, pre-clinical stages, probably related to amyloid toxicity [8]. Moreover, atrophy has been found to be even more prominent in WM than in GM in early stage disease [3]. Consequently, appropriate measures of changes within WM may be valuable biomarkers of neurodegeneration in AD. To date, structural changes of WM have been intensively investigated [9–11], however, there have been no studies investigating functional changes in WM, which presumably link structure and behavioral performance, in the progression towards AD dementia (ADD).

Functional magnetic resonance imaging (fMRI) has been previously used to detect functional alterations arising in AD [12–14] but to date such studies have been almost exclusively focused on GM, with very limited exceptions [15, 16]. FMRI detects changes in MRI images caused by variations in blood oxygenation (blood oxygenation level dependent (BOLD) effects) that in GM correspond to changes in neural activity [17]. Temporal correlation of BOLD signals arising from different GM regions reflects the functional synchronization among these regions, defined as functional connectivity (FC), and has been widely used in neuroimaging studies [18]. By contrast, BOLD signals in WM are expected to be much smaller than those in GM and consequently are excluded from image analyses. However, recently we demonstrated that BOLD fluctuations in WM share common features with those from GM and they correlate significantly with BOLD signals from specific GM regions to which they connect [19, 20]. Relationships between WM tracts and GM regions may be summarized by a functional correlation matrix (FCM) of their pair-wise correlations at rest, while different WM tracts can be inter-related using a similar approach. Those FCs reflect synchronizations of the hemodynamics between WM tracts and GM regions or between two WM tracts, which are hypothesized to be adversely influenced due to biological degeneration, e.g., tissue degeneration and cerebrovascular disorder, occurring during AD evolution.

In this study, we extended these new findings and analyses, originally described in our previous work [19–22], to quantify changes in WM fMRI metrics during the progression to ADD. We measured the differences in the FCMs for WM-GM correlations (FCM_WG_) and WM-WM correlations (FCM_WW_) between a healthy group and each of five participant groups at different stages of cognitive impairments. We also subsequently evaluated the correlations between these FC metrics and neuropsychological measures of cognition and memory. Finally, we explored the use of machine learning to differentiate between the controls and patients at different AD stages to evaluate how well WM FC can predict AD progression.

## Materials and methods

### Participants

Data used in this study were all obtained from the database of the Alzheimer’s Disease Neuroimaging Initiative (ADNI) (adni.loni.usc.edu). The participants included in our study were selected as following. First, the participants must have unprocessed images of both resting state fMRI (rsfMRI) and T1-weighted (T1w) modalities at baseline available in ADNI 2 or ADNI 3. If one participant had multiple datasets, then only the dataset acquired most recently was selected. Second, the participants whose ages were less than 60 or more than 90 years old were excluded. Third, the participants with only multiband rsfMRI were excluded due to the inconsistent acquisition protocol.

The participants were grouped as cognitive normal (CN), significant memory concerns (SMC), early mild cognitive impairments (*e*MCI), mild cognitive impairments (MCI), late MCI (*l*MCI) and ADD. The full criteria for clinical classifications are described in the ADNI manual [23].

### MRI

3T rsfMRI and T1w data, acquired at multiple institutions with the same imaging protocol, were preprocessed using the Data Processing Assistant for Resting State fMRI (DPARSFA) [24], a package based on SPM (https://www.fil.ion.ucl.ac.uk/spm). First, the rsfMRI images were corrected for slice timing and head motion. Twenty-four motion parameters [25] and mean CSF signal were regressed out. The resulting rsfMRI data were filtered (passband = 0.01–0.1Hz), coregistered to MNI space [26], detrended, and then normalized voxel-wise into a time-course with zero mean and unit variance. Next, WM, GM, and cerebrospinal fluid (CSF) were segmented using the T1w images [27] and their tissue probability maps were spatially normalized to the MNI space.

### Calculation of functional correlation matrix (FCM)

The calculations of correlations for each participant were restricted to 48 WM and 82 GM regions of interest (ROIs, listed in Table 1) that were defined by the Eve atlas [28] (21 deep WM tracts in each hemisphere and 6 commissure tracts, see S1 Fig) and PickAtlas [29] (41 Brodmann areas in each hemisphere) and were further constrained within masks generated by thresholding the WM and GM probability maps at 0.8. The preprocessed time-courses were averaged over the voxels within each ROI and for each pair of WM and GM ROIs they were then cross correlated, excluding any time points with large motions (framewise displacement [30] >0.5). The resulting 48x82 correlation coefficients formed an FCM of WM-GM pairs (FCM_WG_). Similarly, the mean time-courses for each pair of WM ROIs were cross correlated and the 48x48 correlation coefficients formed an FCM of WM-WM pairs (FCM_WW_). Meanwhile, we generated FCM of GM-GM pairs (FCM_GG_) to confirm the validity of our processing pipeline. The possible influences of covariates, including age, gender, years of education and acquisition-site, were regressed out from FCM_WG_ or FCM_WW_ element by partialling out procedure via a generalized linear model.

**Table 1:** List of WM and GM ROIs.

White Matter (WM) ROIs		Gray Matter (GM) ROIs	
CST (*l*, *r*):	Corticospinal Tract (*left*, *right*)	BA1 (*l*, *r*):	Primary Somatosensory Cortex 1 (*left*, *right*)
ML (*l*, *r*):	Medial Lemniscus (*left*, *right*)	BA2 (*l*, *r*):	Primary Somatosensory Cortex 2 (*left*, *right*)
ICP (*l*, *r*):	Inferior Cerebellar Peduncle (*left*, *right*)	BA3 (*l*, *r*):	Primary Somatosensory Cortex 3 (*left*, *right*)
SCP (*l*, *r*):	Superior Cerebellar Peduncle (*left*, *right*)	BA4 (*l*, *r*):	Primary Motor Cortex (*left*, *right*)
CP (*l*, *r*):	Cerebral Peduncle (*left*, *right*)	BA5 (*l*, *r*):	Somatosensory Association Cortex (*left*, *right*)
ALIC (*l*, *r*):	Anterior Limb of Internal Capsule (*left*, *right*)	BA6 (*l*, *r*):	Premotor and Supplementary Motor (*left*, *right*)
PLIC (*l*, *r*):	Posterior Limb of Internal Capsule (*left*, *right*)	BA7 (*l*, *r*):	Visuo-Motor Coordination (*left*, *right*)
RLIC (*l*, *r*):	Retrolenticular Limb of Internal Capsule (*left*, *right*)	BA8 (*l*, *r*):	Frontal Eye Fields (*left*, *right*)
ACR (*l*, *r*):	Anterior Corona Radiata (*left*, *right*)	BA9 (*l*, *r*):	Dorsolateral Prefrontal Cortex (*left*, *right*)
SCR (*l*, *r*):	Superior Corona Radiata (*left*, *right*)	BA10 (*l*, *r*):	Anterior Prefrontal Cortex (*left*, *right*)
PCR (*l*, *r*):	Posterior Corona Radiata (*left*, *right*)	BA11 (*l*, *r*):	Orbitofrontal Area (*left*, *right*)
PTR (*l*, *r*):	Posterior Thalamic Radiation (include Optic Radiation) (*left*, *right*)	BA13 (*l*, *r*):	Insular Cortex (*left*, *right*)
SS (*l*, *r*):	Sagittal Stratum (include inferior longitudinal fasciculus and fronto-occipital fasciculus) (*left*, *right*)	BA17 (*l*, *r*):	Primary Visual Cortex (V1) (*left*, *right*)
EC (*l*, *r*):	External Capsule (*left*, *right*)	BA18 (*l*, *r*):	Secondary Visual Cortex (V2) (*left*, *right*)
CGC (*l*, *r*):	Cingulum (Cingulate) (*left*, *right*)	BA19 (*l*, *r*):	Associative Visual Cortex (V3-5) (*left*, *right*)
CGH (*l*, *r*):	Cingulum (Hippocampus) (*left*, *right*)	BA20 (*l*, *r*):	Inferior Temporal Gyrus (*left*, *right*)
FXC (*l*, *r*):	Fornix (Cres) (*left*, *right*)	BA21 (*l*, *r*):	Middle Temporal Gyrus (*left*, *right*)
SLF (*l*, *r*):	Superior Longitudinal Fasciculus (*left*, *right*)	BA22 (*l*, *r*):	Superior Temporal Gyrus (*left*, *right*)
SFO (*l*, *r*):	Superior Fronto-Occipital Fasciculus (*left*, *right*)	BA23 (*l*, *r*):	Ventral Posterior Cingulate Cortex (*left*, *right*)
UF (*l*, *r*):	Uncinate Fasciculus (*left*, *right*)	BA24 (*l*, *r*):	Ventral Anterior Cingulate Cortex (*left*, *right*)
TAP (*l*, *r*):	Tapetum (*left*, *right*)	BA25 (*l*, *r*):	Subgenual Area (*left*, *right*)
MCP:	Middle Cerebellar Peduncle	BA26 (*l*, *r*):	Ectosplenial Portion of Retrosplenial Region (*left*, *right*)
PCT:	Pontine Crossing Tract	BA27 (*l*, *r*):	Piriform Cortex (*left*, *right*)
GCC:	Genu of Corpus Callosum	BA28 (*l*, *r*):	Ventral Entorhinal Cortex (*left*, *right*)
BCC:	Body of Corpus Callosum	BA29 (*l*, *r*):	Retrosplenial Cingulate Cortex (*left*, *right*)
SCC:	Splenium of Corpus Callosum	BA30 (*l*, *r*):	Part of Cingulate Cortex (*left*, *right*)
FX:	Fornix	BA32 (*l*, *r*):	Dorsal Anterior Cingulate Cortex (*left*, *right*)
		BA34 (*l*, *r*):	Dorsal Entorhinal Cortex (*left*, *right*)
		BA35 (*l*, *r*):	Perirhinal Cortex (*left*, *right*)
		BA36 (*l*, *r*):	Ectorhinal Area (*left*, *right*)
		BA37 (*l*, *r*):	Occipitotemporal Area (part of fusiform gyrus and interior temporal gyrus (*left*, *right*)
		BA38 (*l*, *r*):	Temporopolar Area (*left*, *right*)
		BA39 (*l*, *r*):	Angular Gyrus (*left*, *right*)
		BA40 (*l*, *r*):	Supramarginal Gyrus (*left*, *right*)
		BA41 (*l*, *r*):	Auditory Cortex 1 (*left*, *right*)
		BA42 (*l*, *r*):	Auditory Cortex 2 (*left*, *right*)
		BA43 (*l*, *r*):	Primary Gustatory Cortex (*left*, *right*)
		BA44 (*l*, *r*):	Pars Opercularis (*left*, *right*)
		BA45 (*l*, *r*):	Pars Triangularis (*left*, *right*)
		BA46 (*l*, *r*):	Dorsolateral Prefrontal Cortex (*left*, *right*)
		BA47 (*l*, *r*):	Pars Orbitalis (*left*, *right*)

### Neuropsychological test

Neuropsychological scores included the Mini-Mental State Examination (MMSE) score, Clinical Dementia Rating (CDR) global score, CDR Sum of Boxes (CDR-SOB), Global Deterioration Scale (GDS), Functional Assessment Questionnaire (FAQ) total score, Wechsler Memory Scale-Logical Memory II Subscale (WMS-LMII), Alzheimer’s Disease Assessment Scale-Cognitive subscale (ADAS-Cog) and Hachinski scale, which are the most commonly used scores for clinical assessment and AD studies.

### Statistical analysis

The characteristics of the six subject groups were summarized, and the differences among groups were tested by one-way ANOVA or chi-squared test.

The FCMs (FCM_WG_ or FCM_WW_) within each clinical group were averaged to produce a mean matrix (mFCM). Differences in the mFCM values, and the effect sizes of these differences [31] between the CN group and every other group were calculated. To assess the statistical significance of FC difference between groups, the permutation test (10,000 permutations) was conducted for each FCM element across all participants within any two comparison groups. The resulting *P-*values were corrected for multiple comparisons using a false discovery rate (FDR, [32]). Each corrected *P*-value was denoted as *P*_FDR_. To estimate the FC within one WM tract, the so-called WM-tract-wise FC, all the 82 FCM_WG_ elements or 48 FCM_WW_ elements corresponding to this WM ROI were averaged. The mean and standard deviation of each WM-tract-wise FC across participants within each group were then calculated. The WM-tract-wise FCs in the CN group were compared with every non-CN group using unpaired-sample *t*-tests.

To measure the general trend of WM FC as AD progresses, all the elements of each participant’s FCM_WG_ or FCM _WW_ were averaged, defined by overall-FC, and then the group mean and standard deviation of the overall-FC within each clinical group were evaluated, and finally normalized by linear scaling. More specifically, normalization included converting the mean value of CN group into 1 and the mean value of ADD group into 0, calculating the parameters for this linear transformation and then applying the transformation to all the means and standard deviations. On the other hand, the mean and standard deviation of each neuropsychological score within each clinical group were also calculated and normalized by the same linear scaling procedure.

The association between each single FCM element and each neuropsychological score was evaluated by calculating the linear correlation coefficient between the element and the score across all participants. To gauge the integrated correlation of a WM tract’s connectivity with the score, all the correlation coefficients corresponding to the same WM tract were averaged.

To further evaluate the associations between combined FCM elements and each neuropsychological score, a random forest (RF) regression model was trained to predict the score after feature selection from all FCM elements. The value of goodness of fit, R^2^ was calculated based on comparing true and predicted scores. Subjects that did not have any neuropsychological scores were excluded from the regression study.

### Machine learning classification

A support vector machine (SVM) with a radial-basis function kernel was used to classify the CN group and different combinations of groups of impaired subjects (i.e., ADD alone; *l*MCI and ADD; MCI, *l*MCI and ADD; *e*MCI, MCI, *l*MCI and ADD; and SMC, *e*MCI, MCI, *l*MCI and ADD). We used all FCM_WG_ and FCM_WW_ elements as initial features and implemented an RF algorithm to select those features that provided more accurate classifications, a similar procedure to our previous study [33]. In detail, the number of trees for the RF classifier was chosen to be 200 as it was observed that increasing the number of trees further resulted in no significant reduction of classification error. The splitting criterion for RF was based on the GUIDE algorithm [34]. Individual feature importance was computed by measuring how much the predictive accuracy of the RF classifier deteriorates when the feature’s values were randomly permuted [35]. The idea is that altering the value of an important feature will degrade the performance of a classifier. After the importance of each feature obtained individually, the features that did not improve performance at all were at first removed from the set. The remaining features were arranged in descending order of their importance. Features were added sequentially, and classification error was noted for this cumulative feature set. The optimal feature set was taken to be the one which provided the lowest classification error. A similar method was used for feature selection in the case of regression analysis abovementioned. There were 5064 WM FCs in this study, so the features arranged in descending order of importance, were added five at a time sequentially to reduce the computational load. In the case of the classification task, SVM with a radial-basis function (RBF) kernel was optimized with respect to C and Gamma, the two hyper-parameters. C regularized the classifier, and Gamma denoted variance of the RBF kernel and controlled the width of the kernel. Mean squared error of a 10-fold cross-validation (CV) was calculated to measure the classification error. More specifically, the data were split into 10 subsets. The SVM model was trained on 9 subsets and then evaluated on the remaining subsets. This process was repeated 10 times, with a different subset as testing data each time. One error was estimated at each time and the final error was the average of the 10 errors. Also, the penalty involved for misclassification of the disease group versus control group was manually varied so that data imbalance between the groups did not tilt the model accuracy towards one group [36]. Moreover, the receiver-operating characteristic (ROC) analysis was performed and the area under curve (AUC), sensitivity and specificity were noted.

## Results

### Participant characteristics

Table 2 shows characteristics of all 383 participants, grouped into CN (n = 136), SMC (n = 46), *e*MCI (n = 83), MCI (n = 37), *l*MCI (n = 46) and ADD (n = 35), in order of disease severity. Among these groups, no significant differences in age (*P* = 0.93), sex (*P* = 0.22), handedness (*P* = 0.99), years of education (*P* = 0.23), brain volume (*P* = 0.41) or Hachinski scale (*P* = 0.25) were observed. The scanner vendor breakdown, MMSE, CDR global, CDR-SOB, GDS, FAQ, WMS-LMII and ADAS-Cog scores did differ significantly among groups (*P* <0.001).

**Table 2:** Characteristics of participant groups.

Characteristics	CN (n = 136)	SMC (n = 46)	*e*MCI (n = 83)	MCI (n = 37)	*l*MCI (n = 46)	ADD (n = 35)	*P*-value (ANOVA or chi-square)
**Age, mean (SD), y**	74.5 (7.1)	75.3 (5.8)	74.5 (7.0)	74.3 (7.2)	74.6 (6.8)	75.6 (6.8)	0.93
**Female sex, No. (%)**	82(60)	26(57)	42 (51)	15 (41)	21 (46)	16 (46)	0.22
**Handedness, No. (%)**	126 (92)	42 (91)	76 (92)	34 (92)	42 (91)	33 (94)	0.99
**Education, mean (SD), y**	16.8 (2.3)	16.8 (2.6)	16.0 (2.7)	16.3 (2.5)	16.4 (2.9)	16.2 (2.7)	0.23
**Scanner vendor (S:G:P)**	86:27:24	18:15:13	31:11:41	18:9:10	19:6:21	6:3:26	<0.001
Brain volume (SD) X10^5^	10.6 (0.9)	10.7 (0.9)	10.6 (2.2)	10.4 (1.0)	10.4 (1.1)	10.2 (1.2)	0.41
**MMSE score, mean (SD)**	29.1 (1.7)	29.1 (1.0)	27.4 (2.9)	28.0 (1.5)	25.8 (5.6)	22.4 (3.2)	<0.001
**CDR global score, mean (SD)**	0.0 (0.2)	0.1 (0.2)	0.4 (0.3)	0.5 (0.0)	0.6 (0.5)	0.8 (0.2)	<0.001
**CDR-SOB score, mean (SD)**	0.3 (1.0)	0.1 (0.4)	1.7 (2.2)	1.2 (0.8)	2.6 (3.2)	4.7 (1.5)	<0.001
**GDS score, mean (SD)**	0.8 (1.5)	1.3 (1.3)	1.8 (2.0)	1.5 (1.3)	1.8 (2.3)	1.5 (1.3)	<0.001
**FAQ score, mean (SD)**	1.0 (3.7)	0.4 (0.8)	4.2 (6.5)	3.1 (4.4)	6.5 (8.7)	14.6 (6.2)	<0.001
**WMS-LMII score, mean (SD)**	15.4 (3.3)	15.4 (3.0)	12.4 (5.1)	9.2 (3.8)	9.7 (5.0)	4.5 (3.1)	<0.001
**ADAS-Cog score, mean (SD)**	9.6 (4.4)	8.2 (3.1)	12.0 (6.0)	12.3 (3.4)	14.0 (8.3)	22.7 (7.4)	<0.001
**Hachinski scale, mean (SD)**	0.5 (0.7)	0.6 (1.0)	1.0 (1.2)	0.8 (1.2)	0.7 (0.9)	0.9 (0.9)	0.25

Note: S = Siemens; G = GE Medical Systems; P = Philips Medical System and Philips Healthcare.

### WM functional connectivity deficits in progression to AD

**Fig 1:**
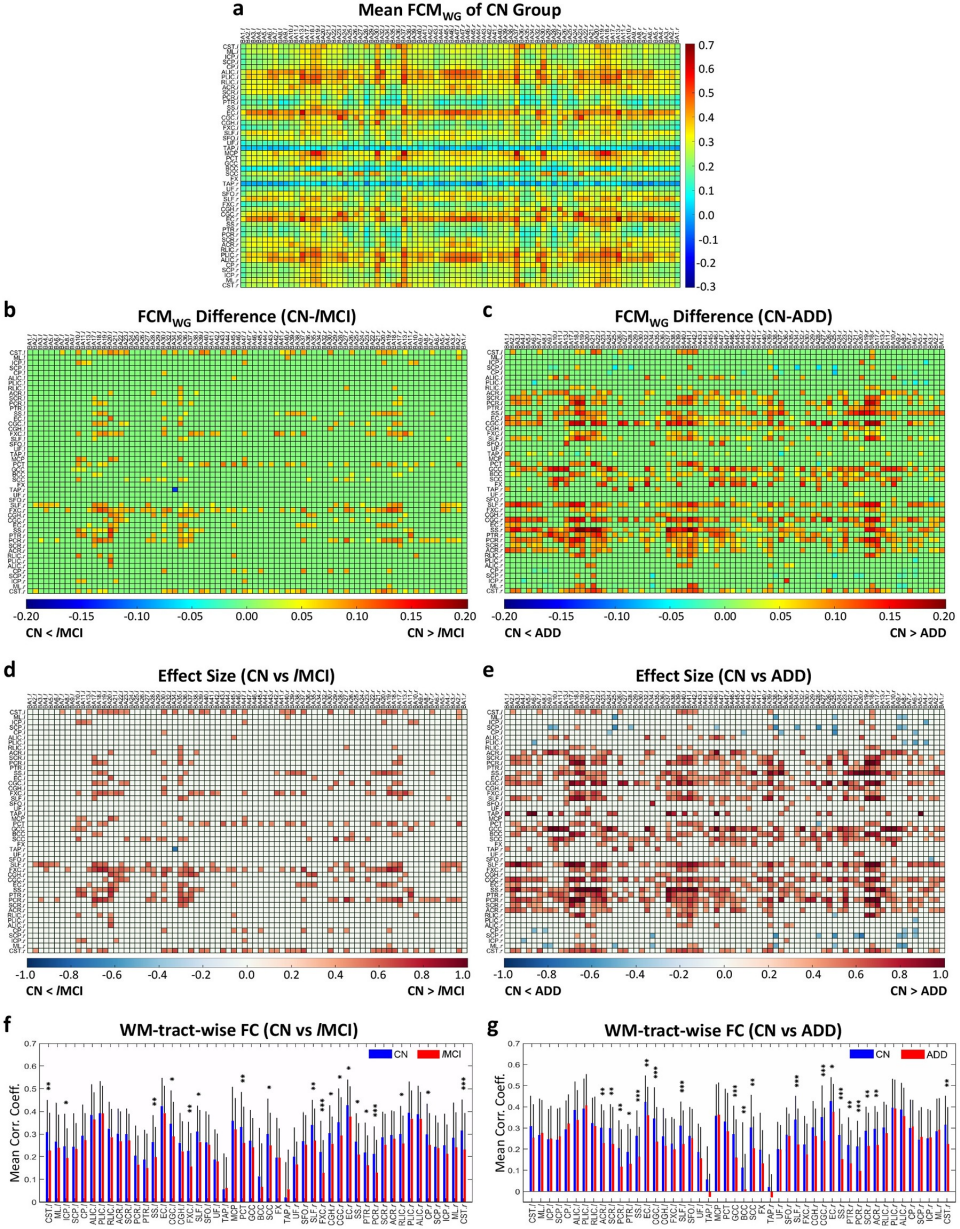
Significant differences in mean FCM_WG_ (mFCMWG) and WM-tract-wise FC between controls (CN) and patients with *l*MCI or ADD. (**a**) mFCM_WG_ of CN group. (**b, c**) Difference of subtracting mFCM_WG_ of *l*MCI (**b**) or ADD (**c**) from mFCM_WG_ of CN group. The *P-*value for each element was derived from permutation-test (10,000 permutations) across all participants within groups, and then adjusted using an FDR. Those elements with *P*_*FDR*_ >0.05 were set to be zero. (**d, e**) Effect size of the mFCM_WG_ difference between CN and *l*MCI (**d**) or ADD (**e**), thresholded by *P*_*FDR*_. (**f, g**) GM-averaged correlation coefficients of WM tracts, i.e., WM-tract-wise FC, in CN group (blue) and *l*MCI group (red) (**f**) and in CN group (blue) and ADD group (red) (**g**). Mean and standard deviation of each WM-tract-wise FC are shown, and * indicates *p* <0.05, ** indicates *p*<0.01 and *** indicates *p*<0.001, calculated by unpaired-sample *t-*test.

Fig 1a presents the mean FCM_WG_ (mFCM_WG_) for CN group, which reveals the general synchronization pattern between whole-brain WM ROIs and GM ROIs during resting state. By comparing this FCM_WG_ to other non-CN groups, we found significant FC decreases (*P*_*FDR*_<0.05) in *l*MCI and ADD patients relative to controls in a number of WM-GM pairs (Fig 1b and 1c). The effect sizes of group difference for those WM-GM pairs were mostly larger than 0.3, shown in Fig 1d and 1e. Furthermore, there were obvious horizontal patterns observed in the difference matrices (Fig 1b and 1c), which appeared to correspond to specific WM tracts. In turn, an in-depth comparison on WM-tract-wise FC, derived from FCM_WG_, between groups was conducted and revealed that the WM-tract-wise FC decreased significantly (*p*<0.05) in the *l*MCI group relative to CN group (Fig 1f) in the following WM tracts: splenium of corpus callosum, pontine crossing fibers, bilateral superior longitudinal fasciculus, bilateral fornix (cres), bilateral cingulum (cingulate), bilateral sagittal stratum, bilateral corticospinal tract, left inferior cerebellar peduncle, right cingulum (hippocampus), right external capsule, right posterior thalamic radiation, right posterior corona radiata, right retrolenticular limb of internal capsule and right cerebral peduncle. Similarly, ADD patients had reduced FC (*p*<0.05) (Fig 1g) in corpus callosum, bilateral superior longitudinal fasciculus, bilateral cingulum (cingulate), bilateral external capsule, bilateral sagittal stratum, bilateral posterior thalamic radiation, bilateral corona radiata, and right corticospinal tract.

**Fig 2:**
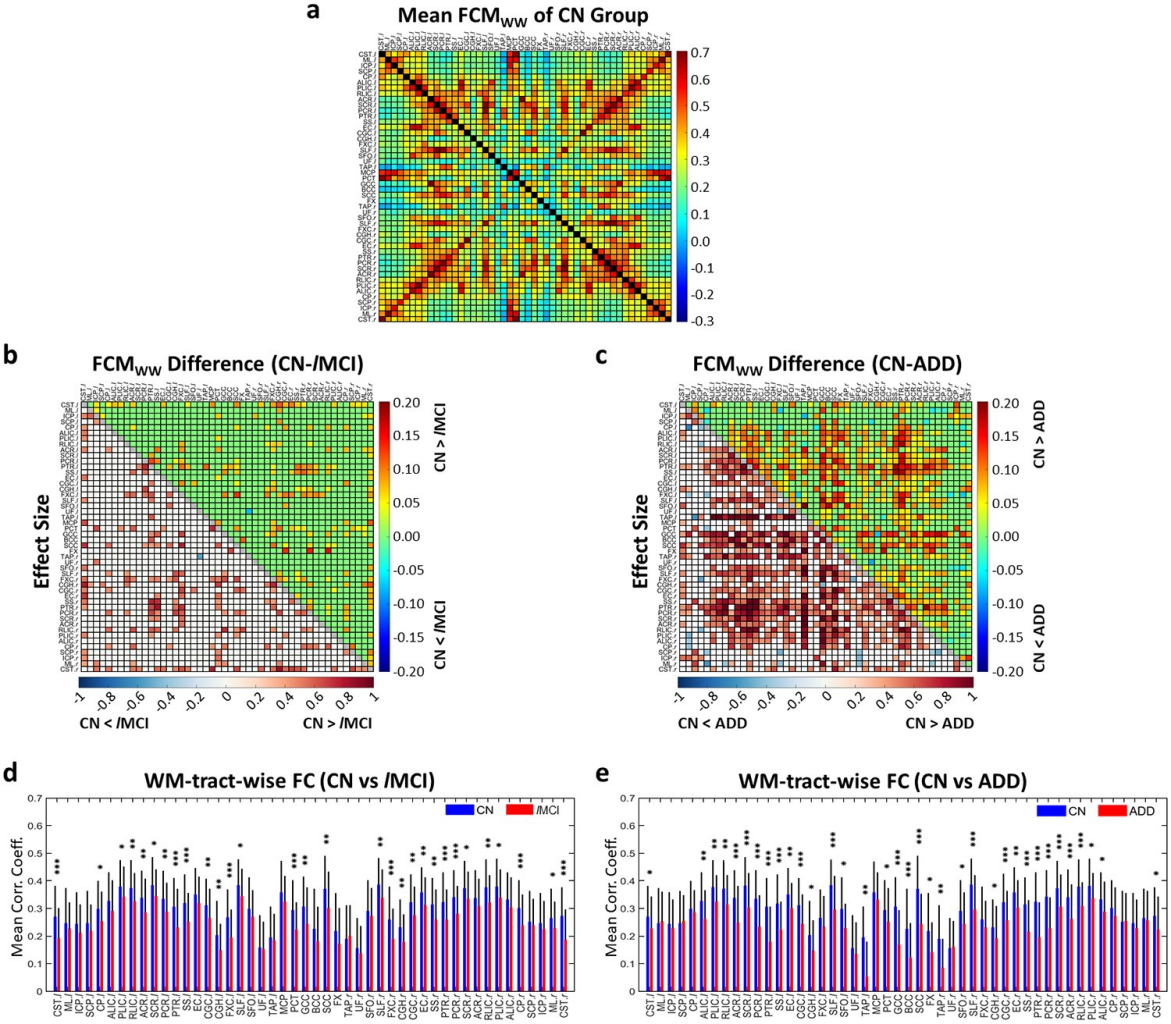
Significant differences in mean FCM_WW_ (mFCMWW) and WM-tract-wise FC between controls (CN) and patients with *l*MCI or ADD. (**a**) mFCM_WW_ of CN group. (**b, c**) Difference of mFCM_WW_ between CN and *l*MCI or ADD (upper triangle) and effect size of the mFCM_WW_ difference (lower triangle). (**d, e**) WM-averaged correlation coefficients of WM tracts, i.e., WM-tract-wise FC, in CN group (blue) and *l*MCI group (red) (**d**) and in CN group (blue) and ADD group (red) (**e**). * indicates *p*< 0.05, ** indicates *p*<0.01 and *** indicates *p*<0.001.

Likewise, Fig 2a exhibits the mean FCM_WW_ (mFCM_WW_) for CN group. The FC significantly decreased (*P*_*FDR*_<0.05) in *l*MCI and ADD patients relative to CN group in a number of WM-WM pairs (upper triangles in Fig 2b and 2c) and the effect sizes of group differences for those WM-WM pairs were mostly greater than 0.3 (lower triangles in Fig 2b and 2c). In terms of WM-tract-wise FC derived from FCM_WW_, significant declines (*p*<0.05) appeared in several tracts in both the *l*MCI and ADD groups relative to the CN group (Fig 2d and 2e), including corpus callosum, superior longitudinal fasciculus, fornix (cres), cingulum (cingulate and hippocampus), external capsule, sagittal stratum, posterior thalamic radiation, corona radiata, internal capsule, and corticospinal tract. There were also a number of WM tracts presenting declined FC only in the ADD groups relative to the CN group, such as fornix, tapetum and superior fronto-occipital fasciculus. The significances of all WM tracts with respect to the decline of WM-tract-wise FC derived from both FCM_WG_ and FCM_WW_ are all summarized in S1 Table.

By contrast, no significant changes in FCM_WG_ or FCM_WW_ between CN and any of the early disease groups (i.e., SMC, *e*MCI and MCI) were observed at the same *P*-value threshold (*P*_*FDR*_<0.05). Moreover, comparisons between mFCM_WW_ and the group mean of GM-GM FCM (mFCM_GG_, S2 Fig) show a generally lower level of WM-WM correlation, with a relative decrease of 25–30% in overall average of FCM for every study group.

### Correlation between WM FC and neuropsychological scores

**Fig 3:**
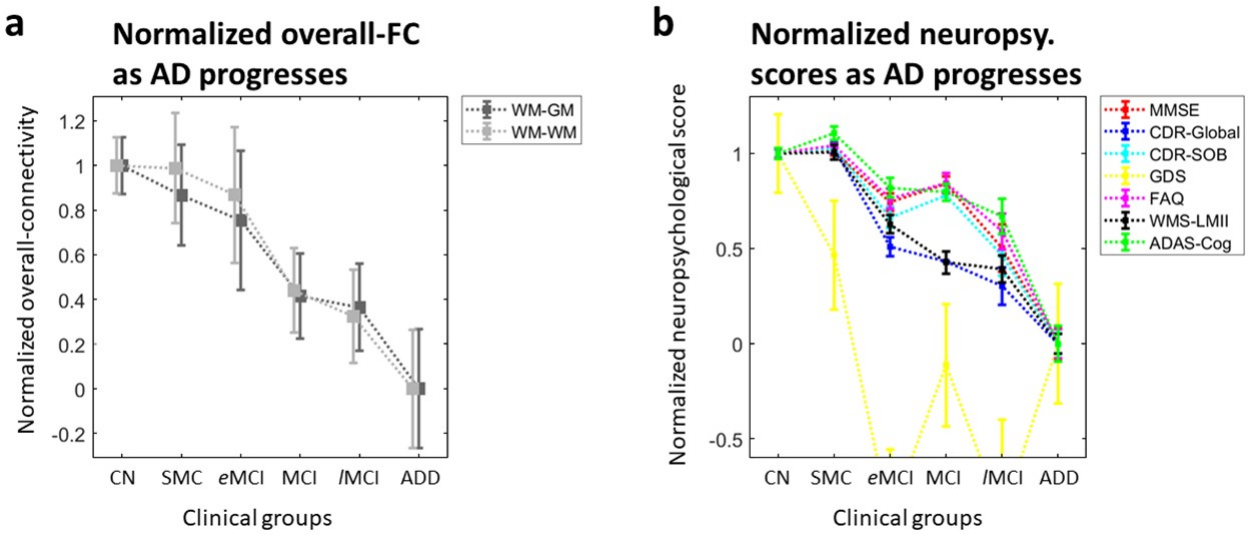
Normalized overall-FC and neuropsychological scores for each clinical group in AD progression. (**a**) Normalized group mean (gray square) and standard deviation of mean (gray bar) of the overall-FC for each clinical group. The six clinical groups are CN, SMC, *e*MCI, MCI, *l*MCI and ADD groups. (**b**) Normalized mean (colored square) and standard deviation (colored bar) of the neuropsychological scores for each clinical group.

The normalized group means of overall-FC presented in Fig 3a decreased gradually as AD progressed, which in general conforms to current hypothetical models of AD evolution [37]. The normalized overall trends in 7 neuropsychological measures across groups (Fig 3b) show a striking similarity with this overall-FC trend.

**Fig 4:**
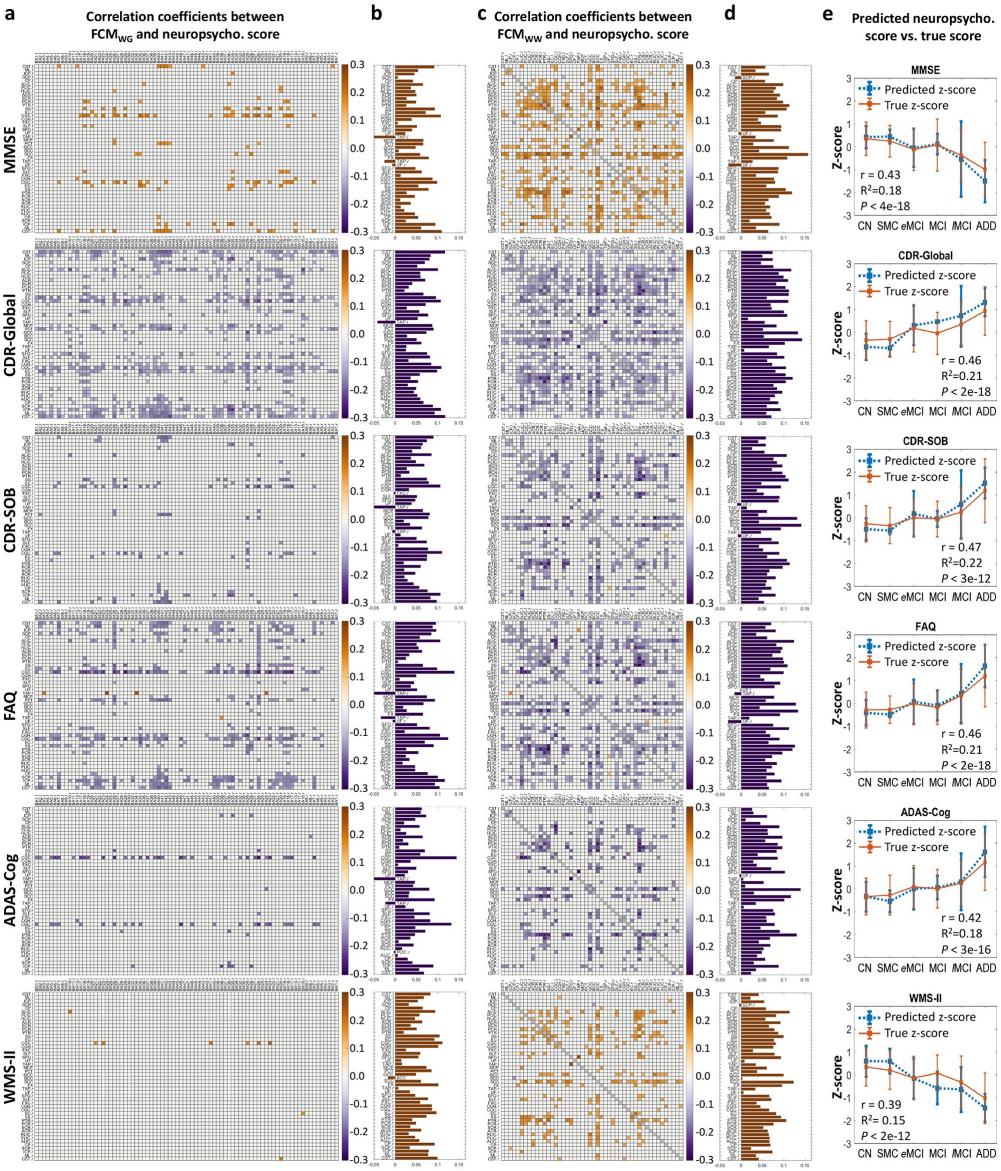
Correlations between WM FC and neuropsychological scores across all subjects. (**a or c**) Matrix of Pearson’s correlation coefficients between single element in FCM_WG_ or FCM_WW_ and MMSE score, CDR-Global score, CDR-SOB score, FAQ score, ADAS-Cog score, or WMS-LMII score. Each correlation coefficient with *P*_*FDR*_ > 0.05 was set to be zero. (**b** or **d**) Average of correlation coefficients along each WM tract in **a** or **c**. See Table 1 for the lists of WM and GM ROIs. (**e**) Group means and standard deviations of Z-scores of true neuropsychological scores and predicted scores using RF regression model with all WM FC as initial features. The r, R^2^ and *P* in each plot are the Pearson’s correlation coefficient between true scores and predicted scores across all subjects, R-square value and *P*-value, respectively.

Fig 4a–4d shows correlation coefficients between elements in FCM_WG_ or FCM_WW_ and neuropsychological scores which are significantly different from zero (*P*_*FDR*_<0.05). No significant correlations were found between any element in FCM and the Hachinski scale or GDS score.

FCM_WG_ and FCM_WW_ elements within bilateral sagittal stratum, bilateral cingulum (cingulate), left cingulum (hippocampus), bilateral fornix (cres), splenium of corpus callosum, bilateral posterior thalamic radiation, and bilateral cerebrospinal tract showed significant positive associations with MMSE scores [38] (Fig 4a–4d), indicating that reduced WM FC corresponds to more severe cognitive impairment.

The CDR-global [39] and CDR-SOB scores are indicators of dementia severity [40]. The sum of negative correlations along WM tracts between FCM and CDR-global was strong in bilateral sagittal stratum, bilateral cingulum (cingulate), bilateral fornix (cres), right superior longitudinal fasciculus, genu and splenium of corpus callosum, fornix, bilateral posterior corona radiata, bilateral posterior thalamic radiation, left anterior limb of internal capsule, and bilateral corticospinal tract (Fig 4a–4d). For the linkage between FCM elements and CDR-SOB score, clear negative correlations were found in bilateral sagittal stratum, bilateral cingulum (cingulate), left fornix (cres), bilateral posterior thalamic radiation, bilateral posterior corona radiata, genu and splenium of corpus callosum, left anterior limb of internal capsule, right retrolenticular limb of internal capsule, and right corticospinal tract.

Negative correlations were dominant in bilateral sagittal stratum, bilateral cingulum (cingulate), bilateral posterior thalamic radiation, bilateral posterior corona radiata, right superior longitudinal fasciculus, left anterior limb of internal capsule, left posterior limb of internal capsule, genu and splenium of corpus callosum between FCM and FAQ [41] that describes the level of performance of daily function activities (Fig 4a–4d).

Bilateral cingulum (cingulate) showed the most significant negative correlations between FCM_WG_ and the ADAS-Cog [42] score, the overall degree of cognitive decline (Fig 4a–4d). Genu and splenium of corpus callosum, fornix, bilateral posterior thalamic radiation, left posterior corona radiata, left cingulum (cingulate), left sagittal stratum, showed significant correlation between FCM_WW_ and ADAS-Cog (Fig 4c and 4d).

Genu and splenium of corpus callosum, bilateral sagittal stratum, left anterior limb of internal capsule, right posterior thalamic radiation, and left cingulum (cingulate) showed stronger positive correlations between FC and the WMS-LMII [43] score, a measure of episodic memory (Fig 4a–4d).

### Correlation between combined WM FCs and neuropsychological scores

The correlation coefficients between the true and RF-predicted scores of ADAS-Cog, CDR-Global, CDR-SOB, FAQ and MMSE were 0.39–0.47 with highly significant *P*-value (<0.001) (Fig 4e). The R^2^ values in Fig 4e indicate that 15%-22% of the variances of those individual scores could be explained by the variance of the combined WM FCs, and vice versa.

### Prediction of AD stages

**Fig 5:**
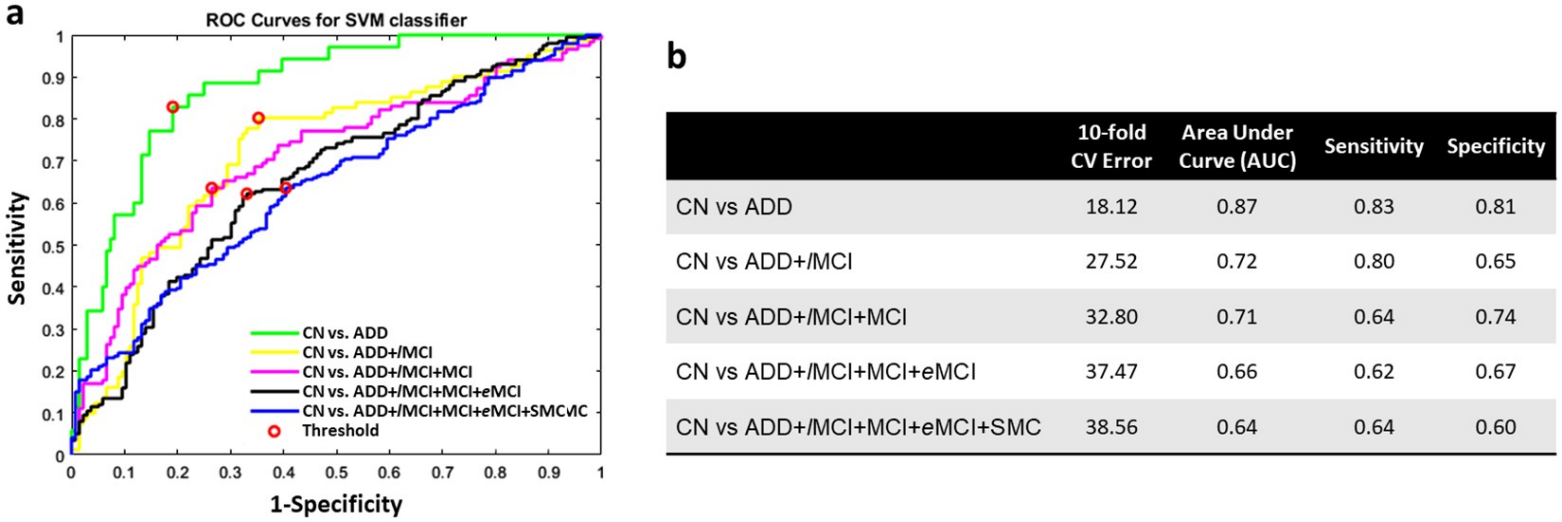
ROC curves of SVM classifications and a summary of their CV errors, AUC, sensitivity and specificity. (**a**) ROC curves of SVM algorithm for distinguishing patients from CN. Different color represents different cumulative group of patients. (b) The errors of 10-fold CV and ROC related indices-AUC, sensitivity and specificity for the classifications.

The performance of the SVM classification using WM FC features was best for distinguishing ADD from CN group with sensitivity of 0.83 and specificity of 0.81 (AUC = 0.87). The performance of prediction reduced monotonically with addition of patients from earlier stages to the ADD group (Fig 5).

## Discussion

In conventional rsfMRI studies, correlations in BOLD signals between GM regions are interpreted as revealing FC. Extending this concept, we investigated the FC between WM and GM regions or between WM regions for 383 ADNI participants from six clinical groups. We mainly found that: 1) *l*MCI and ADD have significant deficits in regional WM FC relative to CN, 2) regional WM FC was significantly associated with neuropsychological scores (i.e., MMSE, CDR, FAQ, ADAS-Cog and WMS-LMII), and 3) the sensitivity and specificity in prediction of late stages of AD using FC as features reach a reasonable range. To the best of our knowledge, this is the first investigation of FC degeneration in WM throughout the evolution of AD pathology. Our findings indicate that FC of WM from MRI may serve as a novel in vivo biomarker to understand functional impairments of WM in AD, especially for the patients at late stages (i.e., *l*MCI and ADD).

Our first batch of findings focused on changes in WM FC as AD progresses. Significant decreases in FC were observed in several localized WM tracts in *l*MCI and ADD patients compared with elderly controls (Figs 1f, 1g, 2d and 2e, or S1 Table). These functional declined WM tracts were mainly projection fibers, association fibers and commissural fibers and have been demonstrated with microstructural degenerations in previous diffusion MRI literature on ADD patients from ADNI [44]. Similar structural degeneration was also observed in brainstem fibers such as corticospinal tract in our study, yet with less consistency across previous studies [44, 45]. Meanwhile, the canonical neuropathological evidence provided by Braak *et al*. [4] also explains the extensive WM hypofunction in the late stages of AD. As to the earlier stages of AD (i.e., SMC, *e*MCI and MCI), although no significant changes (*P*_*FDR*_ < 0.05) were observed in FCM_WG_ or FCM_WW_ in these groups relative to controls, their mean effect sizes were 0.14, 0.14, and 0.19, respectively, suggesting a progression towards non-trivial group differences. Previous studies on brain microstructure reported alterations in a few selected WM regions in MCI participants, involving cingulate bundle, inferior fronto-occipital fasciculus and parahippocampal subgyral fibers [46, 47], where the FC deficits were found in our *l*MCI and ADD groups.

From a pathophysiological perspective, the decreases in WM FC metrics observed in late AD stages may be attributable to GM abnormalities, WM degeneration, metabolic changes and/or cerebrovascular changes. GM abnormalities in AD include the appearance of neurofibrillary/senile plaques, neuronal loss, cell shrinkage, reduced dendritic density and synaptic losses [48, 49]. The consequent neural dysfunction could lead to less engagement of WM in transmission of neural information. WM degeneration during AD progression [50–52] includes demyelination [53] and axonal damage [54], which may also weaken the ability of WM to transfer neural signals between regions. WM degeneration has been reported as a direct consequence of amyloid deposition and tau phosphorylation in GM, and of damage to oligodendrocytes, possibly initiated by ischemia, excitotoxicity, oxidative stress and/or iron overload in AD [55]. Cerebral hypometabolism is found in both MCI and ADD patients throughout limbic structures, involving hippocampal complex, medial thalamus, mammillary bodies and posterior cingulate, yet only in ADD patients in amygdala, temporoparietal and frontal association cortex [56, 57]. Hypometabolism may have direct effects on BOLD signals in GM and WM. Further studies to understand the relationship between the GM changes and decreases in WM connectivity in AD are clearly needed and may reflect a combination of factors including direct causes and effects, comorbidities and mutual dependences.

The second batch of our findings mainly revealed the association between WM FC declines and cognitive impairments, manifest in the WM routes serving limbic network. For example, correlations between WM FC and scores of MMSE, CDR as well as ADAS-Cog were mostly found within sagittal stratum, cingulum, fornix and posterior thalamic radiation, which are the major bundles connecting fronto-occipital pair, cingulate-entorhinal pair, hippocampus-mamillary body pair, thalamus-parietal pair, respectively. Those neural circuits are well-known for playing crucial roles in cognition functioning, such as memory and attention, and suffering from impairment during AD [58–62]. Moreover, the structural damages of those WM tracts were also strongly associated with scores of MMSE, CDR and ADAS-Cog [63, 64]. Similarly, the intrinsic FC between hippocampus and posterior cingulate cortex has been reported to be closely associated with WMS-LM scores in elderly people [65]. In addition, WM tracts in left hemisphere appear to correlate with WMS-LMII scores more widely and strongly (Fig 4a–4d), consistent with previous findings that verbal memory tasks are more sensitive to left hemisphere dysfunction [66] and damage to left temporal lobe has consistently been associated with an impairment of verbal memory [67]. In summary, the consistency between our function-behavior findings and previous structural-behavior evidence suggests that the FC in WM adds a new dimension to connectivity measures and in turn assists in filling the gap of our knowledge between structure in WM and neuropsychological performance.

The performances of our SVM classifications indicate that it is feasible to predict late stages of AD using WM FC only. The performance decreased as patients at earlier stages of AD were included sequentially (Fig 5), just as the sensitivities were lower for detecting FCM difference at earlier stages of AD. However, this does not indicate WM FC is never able to reflect the change in early stages. First, Pearson’s correlation coefficient is the most commonly used metric to measure the FC among GM regions, but its accuracy has been proven to be limited [68]. Given the lower SNR in WM BOLD signals compared to GM signals and the difference in hemodynamic response function between WM and GM [20, 69], the Pearson’s correlation coefficient appears to be even less accurate to represent WM-GM FC. In future, it will be interesting to find better metrics for detecting earlier stages of AD. Second, the FDR we used to conduct multiple comparison turned out to be too strict due to the big size of the matrix (e.g., FCM_WG_: 48x82 = 3936), so the false negative rate might be non-negligible. Moreover, as artificial intelligence developed, such as deep learning algorithms [70], there will be certain method(s) able to distinguish early AD stages from CN. Similarly, we recognize that, while the proposed WM FC metrics allow for determination of neural substrates, currently it is not likely to outperform neuropsychology tests, the primary criteria for staging the disease, in differentiating the study groups. Nevertheless, as our methodology continues evolving, along with advances in MRI technologies, greatly improved performance can be anticipated.

In our analyses, we constrained ROIs to GM or WM only in order to avoid partial volume averaging effects which potentially could overestimate the correlation of time-courses between regions. These correlations of WM with GM or other WM volumes are unlikely caused by drainage effects from adjacent GM because GM drainage occurs outwards, towards the brain surface while deeper WM veins drain inwards to sub-ependymal veins near the lateral ventricles, so there is no direct vascular communication between them [71]. In our analysis we did not regress out global signals because there is growing evidence that they may contain valuable information [72, 73]. Other physiological noises (such as that caused by variations in heart rate and respiration) were, however, regressed out. With these factors in mind, we believe that the WM FC we measured is neither noise effect nor simply a reflection of GM changes.

## Conclusions

The present study indicates that WM FCs 1) decline regionally in *l*MCI and ADD groups relative to a CN group, 2) are significantly related to cognitive scores, and 3) can serve as machine learning features to predict *l*MCI and ADD with a reasonable sensitivity and specificity. These findings suggest the potential of WM FC, which has been largely overlooked to date, as a novel neuroimaging biomarker to assess AD progression.

## Supporting information

S1 Fig Visualization of WM ROIs. The colored WM ROIs are overlaid on the T1 template.
(TIF)

S2 Fig Means of GM-GM and WM-WM functional correlation matrices (mFCM_GG_ and mFCM_WW_, respectively) for each clinical group in AD progression. (**a-f**) mFCM_GG_ (lower triangle) and mFCM_WW_ (upper triangle) for group of CN (**a**), SMC (**b**), *e*MCI (**c**), MCI (**d**), *l*MCI (**e**) and ADD (**f**). Each element in mFCM_GG_ or mFCM_WW_ is the group mean of correlation coefficient of the averaged BOLD time courses between two GM regions or two WM regions. See Table 1 for the list of GM ROIs and WM ROIs.
(TIF)

S1 Table Summary of significance of difference in WM-tract-wise FC between CN group and *l*MCI or ADD group. * *p*<0.05, ** *p*<0.01, *** *p*<0.001.
(DOCX)
