# Supplementary figures and images for: Functional connectivity of white matter as a biomarker of cognitive decline in Alzheimer’s disease
Source: PLoS One. 2020 Oct 16;15(10):e0240513. doi: 10.1371/journal.pone.0240513 (PMC7567362; doi:10.1371/journal.pone.0240513)

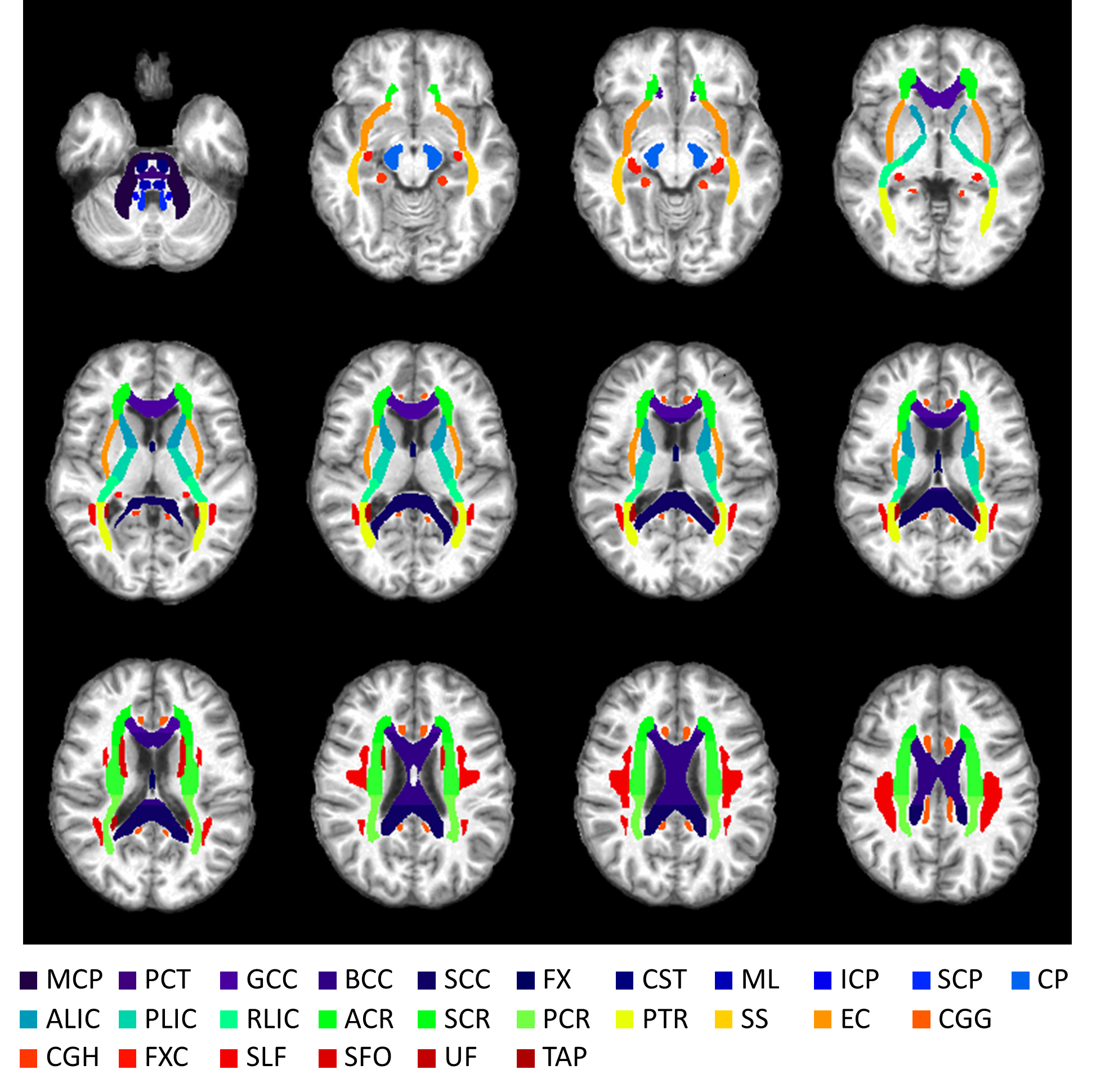

Supplement: S1 Fig — The colored WM ROIs are overlaid on the T1 template. (TIF) [file pone.0240513.s001.tif]

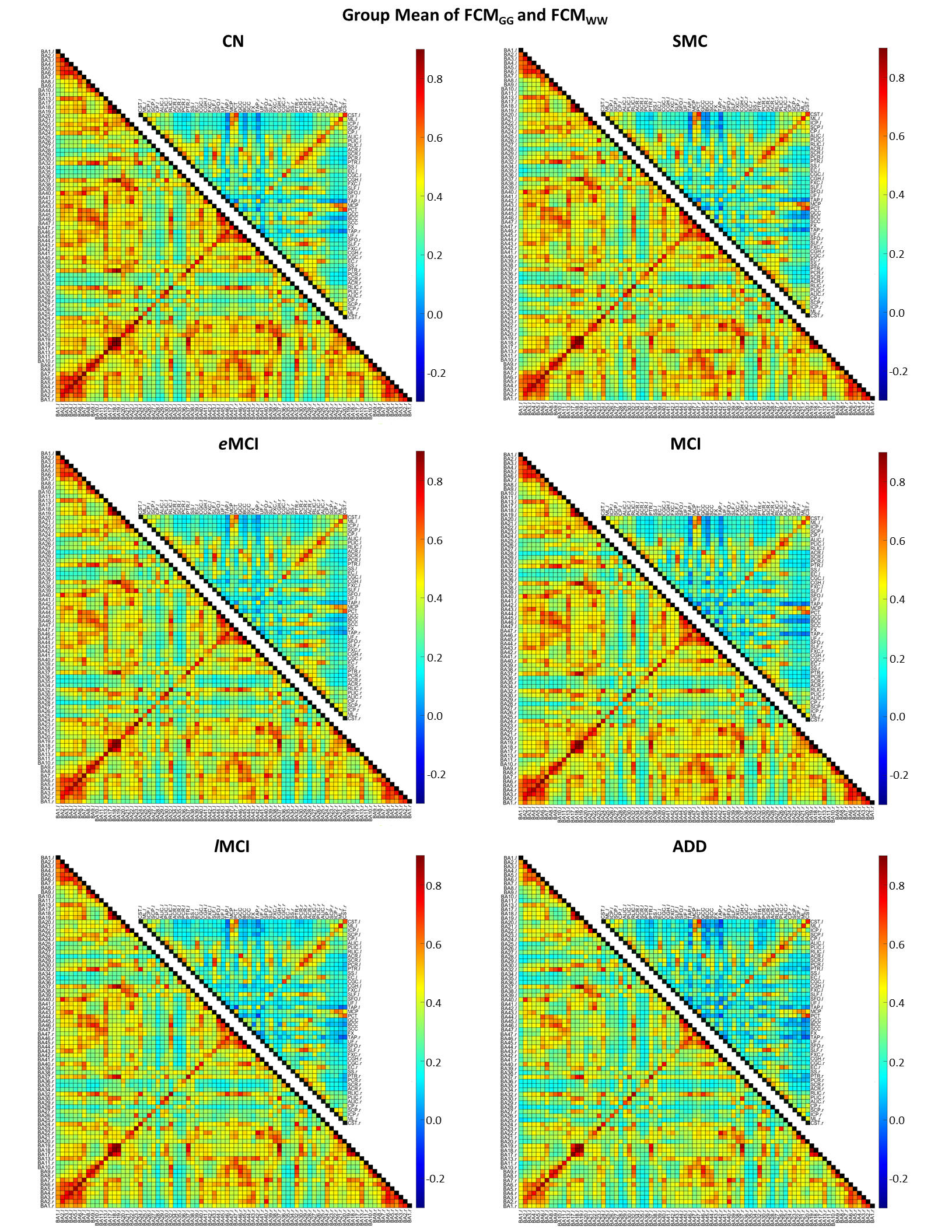

Supplement: S2 Fig — (a-f) mFCMGG (lower triangle) and mFCMWW (upper triangle) for group of CN (a), SMC (b), eMCI (c), MCI (d), lMCI (e) and ADD (f). Each element in mFCMGG or mFCMWW is the group mean of correlation coefficient of the averaged BOLD time courses between two GM regions or two WM regions. See Table 1 for the list of GM ROIs and WM ROIs. (TIF) [file pone.0240513.s002.tif]
