# Supplementary material for: Functional connectivity of white matter as a biomarker of cognitive decline in Alzheimer’s disease
Source: PLoS One. 2020 Oct 16;15(10):e0240513. doi: 10.1371/journal.pone.0240513 (PMC7567362; doi:10.1371/journal.pone.0240513)
Supplement: S1 Table — * p<0.05, ** p<0.01, *** p<0.001. (DOCX) [file pone.0240513.s003.docx]

|  | WM tracts | |  | WM-GM | | WM-WM | |
| --- | --- | --- | --- | --- | --- | --- | --- |
|  | **Abbr.** | **Full name** |  | ***l*MCI < CN** | **ADD < CN** | ***l*MCI < CN** | **ADD < CN** |
| **Brainstem** | CST | Corticospinal Tract | *l* | ** |  | *** | * |
|  |  |  | *r* | *** | ** | *** | * |
|  | ML | Medial Lemniscus | *l* |  |  |  |  |
|  |  |  | *r* |  |  | * |  |
|  | ICP | Inf. Cerebellar Peduncle | *l* | * |  |  |  |
|  |  |  | *r* |  |  |  |  |
|  | MCP | Middle Cerebellar Peduncle |  |  |  |  |  |
|  | SCP | Sup. Cerebellar Peduncle | *l* |  |  |  |  |
|  |  |  | *r* |  |  |  |  |
|  | PCT | Pontine Crossing Tract |  | ** |  | *** | * |
| **Projection** | CP | Cerebral Peduncle | *l* |  |  | * |  |
|  |  |  | *r* | * |  | *** |  |
|  | ALIC | Ant. Limb of Internal Capsule | *l* |  |  |  | ** |
|  |  |  | *r* |  |  |  | * |
|  | PLIC | Post. Limb of Internal Capsule | *l* |  |  | * | ** |
|  |  |  | *r* |  |  | * | * |
|  | RLIC | Retrolenticular Limb of Internal Capsule | *l* |  |  | ** | ** |
|  |  |  | *r* | * |  | ** | *** |
|  | ACR | Ant. Corona Radiata | *l* |  | ** | ** | *** |
|  |  |  | *r* |  | ** |  | *** |
|  | SCR | Sup. Corona Radiata | *l* |  | ** | * | *** |
|  |  |  | *r* |  | ** | * | *** |
|  | PCR | Post. Corona Radiata | *l* |  | ** | ** | *** |
|  |  |  | *r* | *** | *** | *** | *** |
|  | PTR | Post. Thalamic Radiation (include OR) | *l* |  | * | *** | *** |
|  |  |  | *r* | * | ** | *** | *** |
| **Association** | SS | Sagittal Stratum (inf. longitudinal fasciculus and fronto-occipital fasciculus) | *l* | ** | *** | *** | *** |
|  |  |  | *r* | * | *** | ** | *** |
|  | EC | External Capsule | *l* |  | ** |  | ** |
|  |  |  | *r* | * | * | ** | ** |
|  | CGC | Cingulum (Cingulate) | *l* | * | *** | ** | *** |
|  |  |  | *r* | * | *** | ** | *** |
|  | CGH | Cingulum (Hippocampus) | *l* |  |  | ** | * |
|  |  |  | *r* | * |  | ** | * |
|  | FXC | Fornix (Cres) | *l* | ** |  | *** |  |
|  |  |  | *r* | *** |  | *** |  |
|  | SLF | Sup. Longitudinal Fasciculus | *l* | * | *** | * | *** |
|  |  |  | *r* | ** | *** | ** | *** |
|  | SFO | Sup. Fronto-Occipital Fasciculus | *l* |  |  |  | * |
|  |  |  | *r* |  |  |  | * |
|  | UF | Uncinate Fasciculus | *l* |  |  |  |  |
|  |  |  | *r* |  |  |  |  |
|  | FX | Fornix |  |  |  |  | * |
| **Commissure** | GCC | Genu of Corpus Callosum |  |  | *** | ** | *** |
|  | BCC | Body of Corpus Callosum |  |  | ** |  | *** |
|  | SCC | Splenium of Corpus Callosum |  | * | ** | ** | *** |
|  | TAP | Tapetum | *l* |  |  |  | ** |
|  |  |  | *r* |  |  |  | ** |
